# Security Privacy and Policy for Cryptographic Based Electronic Medical Information System

**DOI:** 10.3390/s21030713

**Published:** 2021-01-21

**Authors:** Hsuan-Yu Chen, Zhen-Yu Wu, Tzer-Long Chen, Yao-Min Huang, Chia-Hui Liu

**Affiliations:** 1 National Defense Medical Center, Department of Radiology, Tri-Service General Hospital, Taipei 10086, Taiwan; penguin0916@livemail.tw; 2 Department of Information Management, National Penghu University of Science and Technology, Penghu 880011, Taiwan; zywu@gms.npu.edu.tw; 3 Department of Finance, Providence University, Taichung 43301, Taiwan; tlchen1976@pu.edu.tw; 4 Department of Management Sciences, National Chiao Tung University, Hsinchu 30010, Taiwan; yaomin.ms07g@nctu.edu.tw; 5 Department of Applied Mathematics, Chinese Culture University, Taipei 11114, Taiwan

**Keywords:** privacy, electronic medical record, electronic prescriptions, electronic medical information

## Abstract

With the development of the internet, applications have become complicated, and the relevant technology has diversified. Compared with medical applications, the significance of information technology has been expanding to include clinical auxiliary functions of medical information. This includes electronic medical records, electronic prescriptions, medical information systems, etc. Although research on the data processing structure and format of various related systems is becoming mature, the integration is insufficient. An integrated medical information system with security policy and privacy protection, which combines e-patient records, e-prescriptions, modified smart cards, and fingerprint identification systems, and applies proxy signature and group signature, is proposed in this study. This system effectively applies and saves medical resources—satisfying the mobility of medical records, presenting the function, and security of medicine collection, and avoiding medical conflicts and profiteering to further acquire the maximum effectiveness with the least resources. In this way, this medical information system may be developed into a comprehensive function that eliminates the transmission of manual documents and maintains the safety of patient medical information. It can improve the quality of medical care and indispensable infrastructure for medical management.

## 1. Introduction

For several years of network development, applications have become complicated, and the relevant technology is becoming more diversified. Digitalization in daily life has become a major trend in modern technology development. Common e-commerce, e-medical treatment, e-banking, g-government, and online community applications are included in this network. Relative to medical applications, the meaning of information technology lies in expanding the clinical facilitating function of medical information. The technology covers health insurance IC card devices [1], digital certificates [2] and signatures [3], electronic medical records [4], and electronic prescriptions [5].

The introduction of information technology to medical systems presents an improvement to medical management performance. However, the development and maintenance costs for medical information systems are large that it is simply adopted in large medical centers. Besides, it is merely one-way information feed-in, rather than interactive information feedback, such as assisting medical personnel in teaching, inquiry, aid, and alert with built-in professional medical knowledge. Research on the data structure of health insurance IC cards and digital certificates, electronic medical records, and the data format of electronic prescriptions are out of date. However, the integration of cross-medical institution electronic medical record format [6], cross-department secure patient record information exchange agreement [7], telemedicine, and caregiver authentication mechanisms still require improvement. These are research on cross-medical institution electronic medical record format exchange agreement and medical information system transfer format.

The reinforcement and integration of medical information systems could assist in the promotion of medical quality and efficiency, where the integration of function and technology is the key to implement medical information.

(1) Reinforce the function of health insurance IC cards and the compatibility with integrated medical information systems: The generally used health insurance IC cards are wafer cards with small memory capacity, bad computing function, and not being able to support digital signature or encryption/decryption requirements in actual applications that it is not convenient for the verification and acquisition of electronic medical records, prescriptions, and examination forms.
(2) Establish an electronic prescription system with complete functions and the integration with medical information systems: With the rapid development of electronic medical records, prescriptions are more often electronic. In terms of current medical systems and health insurance systems, the assistance of digital signature allows pharmacies and patients to verify the correctness, integrity, and non-repudiation of electronic prescriptions; meanwhile, pharmacies could complete the health insurance payment report through online verification mechanisms.
(3) Protect patients’ and doctors’ privacy: Confidentiality in medical practice is the basic element to establish a good doctor–patient relationship. Patients have the right to request the confidentiality of personal medical information, and doctors have an obligation to respect patients’ medical privacy. In the essence of law, privacy is a limited right that is passively restricted to balancing the conflict among public health benefits, third-party benefits, and personal privacy benefits. Moreover, under the current medical system (where referrals, consultations, and health insurance IC cards, medical division, and medical teams are the trend), there is also a major challenge to maintain patients’ privacy. In this case, authorized access to patients’ medical records could implement the protection of both doctors’ and patients’ privacy. An integrated medical information system with security policy and privacy protection, which combines e-patient records, e-prescriptions, modified smart cards, and fingerprint identification systems and applies proxy signature and group signature, is proposed in this study.

## 2. Related Works

An integrated medical information system combines various computer science technologies, e.g., smart cards, electronic medical records, public-key cryptosystems, digital signature. The digital signature could be extended into a group signature or proxy signature and applied to different research fields.

### 2.1. Smart Cards

Smart cards were a plastic card with ISO standards and embedded an IC chip. By reading the data on the chip, it presented various functions of memory, identification, encryption/decryption, and transmission. Since a smart card provides a simple computation function of a computer, the card could be used for storing personal data, e.g., medicine, identity information, key, public certificate, electronic medical records, and prescriptions, and various personal diagnostic records. The transmission and encryption of data are processed through the central processing unit to become safer. The data record could be stored with the memory and eventually transmitted with an import/export interface through a reader. In the medical information system proposed in this study, healthcare certification IC cards and health insurance IC cards derived from smart cards are applied. A healthcare certification IC card, also named a doctor card, used in current medical institutions contains a medical personnel card, doctor card, and the additional card of the medical institution. These three cards allow doctors or medical personnel in a medical institution to develop the functions. Each card presents distinct functions. Ones with medical personnel cards could view medical data, but not patients’ personal data. Those with doctor cards, on the other hand, could view patients’ data and medical records [8,9].

A doctor card plays a critical role in the system. A doctor card represents a doctor. The group signature key in a doctor card is required for retrieving electronic medical records in the system using process, and the signature with a doctor card is necessary for medicine collection and insurance. In this case, the doctor with the signature could be found out for medical malpractice claims. Health insurance IC cards were formally used in 2004. Past health insurance paper cards were then gradually eliminated. To cope with this trend, the type of health insurance certificates is often digital document [10].

A health insurance IC card could be regarded as a national health insurance IC card with complete functions and multiple usages. In addition to replacing original health insurance paper cards, other data with health insurance certificates, such as children’s health booklets, maternal health booklets, and catastrophic illness cards, are also integrated into the card. In this case, a patient could simply take the health insurance IC card, regardless of identity, to see a doctor.

### 2.2. Electronic Medical Records

Medical records are medical business document generated in the process of various examinations, diagnoses, treatment, and care when the staff in a medical institution engaging in medical businesses, including medical records, certificate agreement, examination records, and nursing records. Due to distinct storing methods of hospitals, medical records are scattered and not easily shared and exchanged to result in bad efficiency and waste of medical resources. To improve such a situation, paper medical records are gradually developed into electronic medical records for conveniently integrating and updating data, as well as circulating in various medical institutions when necessary [11]. Several hospitals are currently making efforts toward electronic medical records. The US Computer-Based Patient Record Institute (CPRI) describes electronic medical records as “electronically storing individual health conditions and medical care information in the life” to replace paper medical records as the major medical care records [12].

The Institute of Medicine (IOM) further indicated that electronic medical records should be able to provide complete and accurate data, as well as clinical decision-making and medical research. The US Medical Records Institute (MRI) defined electronic medical records as the electronic information of individual health state and medical care in the life, and the development of electronic medical record system was divided into five stages, namely, automated medical record (AMR), computerized medical record (CMR), provider-based electronic medical record (EMR), and electronic health record (EHR) [10]. The Institute of Medicine (IOM), United States National Academy of Sciences, pointed out six different data formats, including text, graphics, and images, numerical, sound, and full-motion video [13]. An electronic medical record system, therefore, was the mechanism used to store data. IOM explained that the mechanism was the system composed of personnel, data, rules, process, processing, storing equipment, communication, and technological support to provide the acquisition, use, storage, and extraction of medical records.

The boom of information technology in past years has resulted in various cloud application services, with lots of convenient and advantages, such as concentration management, saving storage space, and convenience in sharing with other users. Nevertheless, such convenience has resulted in great problems and possible causes information loss or patients’ privacy being stolen and even abused. Hence, using electronic medical records in the cloud does involve certain risks. For example, Google Health builds its e-medical records on the cloud environment, providing users with self-management service. However, this convenience causes a big problem. Because in its privacy policy, Google Health indicates that the user can permit others (e.g., their doctors or pharmacy) to share their medical records and add the information to their medical records (e.g., test results). Although the users can withdraw the permission anytime, the previously authorized individuals or institutions may have already viewed the medical information or even kept copies of the information, which may cause the loss of information or the theft or even abuse of the patient’s privacy. When the people with administrator authority in Google can view the e-medical records, storing e-medical records in the cloud poses certain risks.

For this reason, this study store electronic medical records in the Bureau of National Health Insurance; various medical businesses are contracted by the Bureau of National Health Insurance, and the secure and efficient encryption technology is used for the transmission to protect patients’ medical records.

### 2.3. Digital Signature

The digital signature was originated from cryptography, functioning to provide the mechanism to sign text information and ensure the data being completely and securely transmitted to the destination [14,15]. It is generally used with a one-way hash function, which is a kind of mathematical algorithm that acquires an output value with constant length by inputting text information with any length. Hash function mainly functions to avoid the third-party deriving the input value from the output value [16].

The descriptions above briefly explain the generation method of a digital signature. With a public-key system as the basis, the transmitter first uses a one-way hash function to transform electronic documents into text information with constant length, called a message digest. A personal, secret key is then used to sign the message digest to generate a digital signature. After the recipient receives the message and signature, the transmitter’s public key is used for verifying the signature integrity and confirm the message computed by hash function conforming to the content. Inconsistent content reveals the received information being tampered with. On the contrary, consistent contents show that the received message is a valid document. Accordingly, the user could confirm the integrity and security of data transmission.

(1) Group Signature: D. Chaum and E. van Heyst proposed the idea of group signature in 1991, as the application of digital signature. The group members, as a group, anonymously sign for the message [17]. When there is a dispute, a specific just third-party is responsible for checking the signature and tracing the real signer. Group signature is normally used for a group announcing message. The signature is generated by any members in the group using a personal, secret key, and the verification merely requires a public group key. Logically, group signature is classified as the signature mechanism with multiple secret keys to verify a public key [18]. As the example of medical record transmission, a patient might register different departments for the same outpatient clinic time that errors might appear in the medical record transmission process. In this case, a group signature could be used for checking the verifiability of departments and establishing the non-linkability of a signer; or, when there is a dispute, a creditable third-party could trace the signer’s identity with the secret key.
(2) Proxy Signature: Mambo et al. proposed proxy signature in 1996 [19], including the operation mechanism of the original signer, proxy signer, and verifier. Under the authorization of the original signer, a proxy signer could make the decision. The authorization model is divided into authorization, partial authorization, and letter of authorization, where the letter of authorization is the most common one [20,21]. A letter of authorization specifies the original signer’s identification code, the proxy signer’s identification code, and the term. It could be used for collecting medicine so that disabled patients or those not being able to collect medicine could authorize an agent, with signature authorization, to collect medicine.

### 2.4. Electronic Medical Records System

There was already some security protocols that protect privacy within Medical Information System. In 2018, C. T. Li et al. proposed an enhanced secure protocol for cloud-based telemedical information system [22]. It could provide user anonymity and unlinkability. Their authentication scheme included four phases: (1) Healthcare center upload phase, (2) patient data upload phase, (3) treatment phase, and (4) check-up phase. Through the security, an analysis verified that their scheme cost fewer computation costs against various known attacks. The authentication scheme for telemedical information system-based cloud environment could ensure privacy preservation. Y. Wang et al. proposed the cloud-based e-health record system in 2019 [23]. For the centralization of a cloud-based system that exposed threats to security, they used searchable encryption and proxy-re-encryption to construct their e-health record information sharing system, based on Consortium blockchain.

The K-Anonymity mechanism was proposed to protect hospital internal information systems and electronic medical records publishing in 2018 [24]. K. Fan et al. [25] designed a blockchain-based medical information system, MedBlock, to manage the patients’ information. The distributed ledger of MedBlock system has an efficient access control method and secure authority. The MedBlock system can reduce the large energy consumption and network congestion. O. Enaizan et al. proposed a framework based on a multi-criteria standpoint in Malaysia. Using the Unified theory of acceptance and use of technology 2 (UTAUT2) clarifies the construction between individual, privacy, and EMR system acceptance [26]. The research results show five factors that have a positive effect of EMR acceptance. The five factors includes: Data integrity, confidentiality, non-repudiation, facilitating conditions, and effort expectancy. With the development of information technology, Y. Cao et al. proposed a hybrid blockchain-based secure sharing scheme [27]. They also use Hyperledger Caliper, which is a blockchain benchmark that estimates performance.

## 3. Results

The proposed Integrated Medical Information System (IMIS), including the architecture, physical mechanism, and execution process, are introduced in this section. IMIS integrates e-patient records and e-prescriptions to simplify the originally complicated and time-consuming medical process, e.g., diagnosis, inspection, medicine collection, emergency, and insurance payment. It even applies cryptography, e.g., encryption/decryption and digital signatures, to protect patients’ and doctors’ privacy, and prevent illegal benefit acquisition. The parameters’ definitions are shown in Table 1.

**Figure 1:**
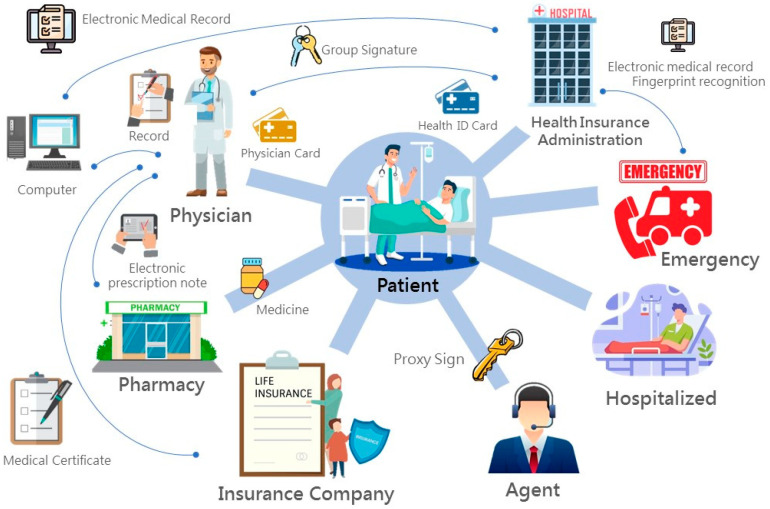
The Integrated Medical Information System (IMIS) process.

The architecture and operation process of IMIS are shown in Figure 1. The entire mechanism could be divided into the registration phase, diagnosis phase, collecting medicine phase. The physical mechanism contains insurers, the Bureau of National Health Insurance, pharmacies, patients, doctors, and agents. The Bureau of National Health Insurance is responsible for national medical businesses, covering from doctors to patients’ medicine collection. A patient is first offered a national health insurance IC card used for medical institutions; doctors and pharmacies are provided consultation and medicine authentication, including collecting, storing, and updating patients’ electronic medical records and prescriptions; and an agent verifies the qualification to collect medicine.

### 3.1. Registration Phase

In the initial step, it is necessary to apply for an anonymous national health insurance IC card from the Bureau of National Health Insurance (NHI) to protect patients’ (P) privacy. The secret key and relevant information are stored in the national health insurance IC card. A patient’s request *R_P_* for the application is shown as Equation (1). The content covers the detailed items for applying for health insurance *Req*, the agent’s public key *PK_AP_* required for outpatient, and the signature made with *Req* and *PK_AP_* through personal, secret key *SK_P_*. The key certificate *Cert_P_* is also included and transmitted to the Bureau of National Health Insurance through a secure channel.

(1) $$P\to NHI:R_{P}=\{Req,\;PK_{AP},\;Sig_{SK_{P}}(Req,\;PK_{AP}),\;Cert_{P}\}$$

**Figure 2:**
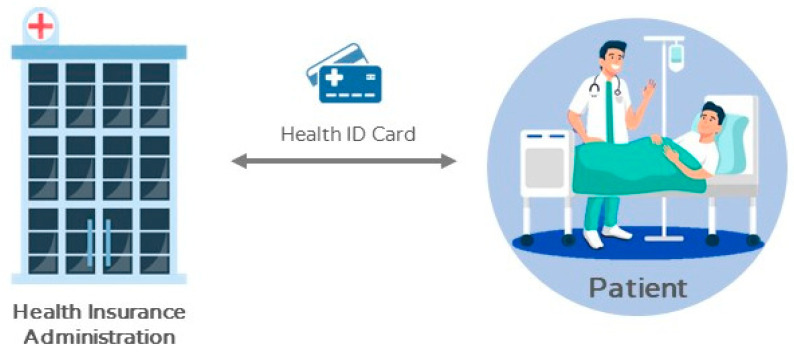
Patients registering the Bureau of National Health Insurance.

After receiving *R_P_*, the Bureau of National Health Insurance could apply the public key information *PK_P_* in *Cert_P_* to verify the correctness of the signature $Sig_{SK_{P}}(Req,\;PK_{AP})$. When it is correct, a serial number *PID_P_* is given to the patient. *PID_P_* could be a pseudo of the patient. The *ID* would be used for seeing a doctor, collecting medicine, or applying for payment. Except for the Bureau of National Health Insurance, no one could acquire the patient’s real name from other information, including keys or medical record data. What is more, *Data* is the certificate of a patient paying insurance fees to prove that the patient is legal and is able to pay medical expenses. Finally, Equation (2) presents the agent’s public key *PK_AP_* transmitted by the patient, the patient’s public key *PK_P_*, and the public key certificate *PCert_P_* generated by personal, secret key *SK_NHI_*. The national health insurance IC card stored with data is given to the patient. In addition to the public key certificate *PCert_P_*, the patient needs to save personal, secret key *SK_P_* in the national health insurance IC card, as shown in Figure 2.

(2) $$NHI\to P:PCert_{P}=\{PID_{P},\;Data,\;PK_{AP},\;PK_{P},\;Sig_{SK_{NHI}}(PK_{AP},\;PK_{P})\}$$

**Figure 3:**
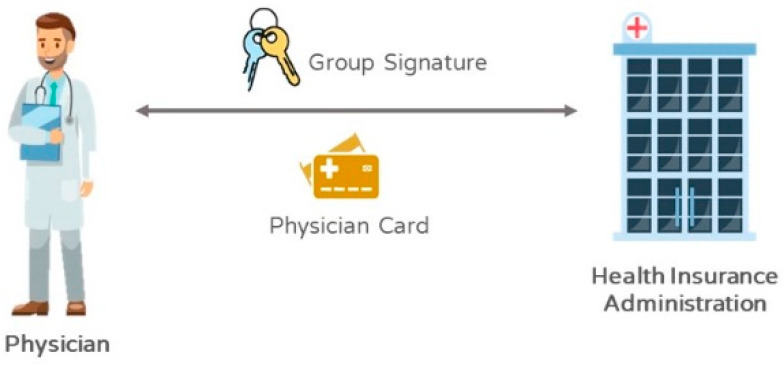
Doctors registering within the Bureau of National Health Insurance.

Regarding the protection of a doctor’s privacy, a doctor (DR) must apply for a doctor card and personal group signature secret key from the Bureau of National Health Insurance, and save the group signature secret key in the doctor card, as shown in Figure 3. In addition to the Bureau of National Health Insurance being able to know the doctor with the signature, anyone could simply utilize a group signature public key *PK_GK_* to verify the correctness and integrity, but not knowing the owner of the signature. It satisfies the characteristics of anonymity and privacy protection. Accordingly, a doctor with the identity *ID_D_* for the secret key *SK_D_* signature, the key certificate *Cert_D_* is transmitted to the Bureau of National Health Insurance through a secure channel for applying for the group signature *R_D_*, as shown in Equation (3).

(3) $$DR\to NHI:R_{D}=\{ID_{D},\;Sig_{SK_{D}}(ID_{D}),\;Cert_{D}\}$$

After the Bureau of National Health Insurance verifies the signature correctness, the *Master Key* (*MK*) of the group signature is combined with the doctor’s *ID_D_* to generate the group signature secret key *K_D_* through *F_GK_* for the doctor, as shown in Equation (4). Finally, the doctor card stored with the data is given to the doctor to complete the signature key.

(4) $$NHI\to DR:F_{GK}(MK,\;ID_{D})=K_{D}$$

Furthermore, IMIS presents the function of an agent (A) assisting patients in collecting medicine. When necessary, a patient and an agent, after the Bureau of National Health Insurance confirms the agent’s legality, could apply the patient’s secret key *SK_P_* and the agent’s secret key *SK_A_* to generate agent’s signature secret key *SK_Ap_* with *F_A_*, as shown in Equation (5). The agent’s signature secret key *SK_Ap_* is stored in the national health insurance IC card.

(5) $$P↔A:F_{A}(SK_{P},\;SK_{A})=SK_{Ap}$$

### 3.2. Diagnosis Phase

#### 3.2.1. Outpatient Clinic

A patient at this phase would carry the national health insurance IC card to see a doctor. The process is described below. First, the patient inserts the national health insurance IC card into a registration machine in the hospital and uses a personal, secret key *SK_P_* for signing the recent signature *TS* on the computer. The hospital would inspect the legality of the patient’s health insurance payment data *Data*. In the outpatient, the doctor and the patient should insert the doctor card and the national health insurance IC card to the reader and encrypt (*E*) the signature *TS* and the public key certificate *PCert_P_* with the public key*PK_NHI_* of the Bureau of National Health Insurance, as shown in Equation (6).

(6) $$P\to DR:E_{PK_{NHI}}(TS,\;Sig_{SK_{P}}(TS),\;PCert_{P})$$

The machine connects to the system of the Bureau of National Health Insurance, which would decrypt (*D*) with the secret key *SK_NHI_* and the patient’s public key *PK_P_* to resolve *PK_NHI_* and authenticate the correctness of the signature $Sig_{SK_{P}}(TS)$, as shown in Equation (7).

(7) $$DR↔NHI:D_{SK_{NHI}}(E_{PK_{NHI}}(TS,\;Sig_{SK_{P}}(TS),\;PCert_{P}))$$

Once the authentication is correct, and the patient is confirmed, the authentication of the group signature public key would be saved in the machine in the Bureau of National Health Insurance to ensure the investigation for medical malpractice claims. The patient’s basic data, medical records, and the recent diagnosis records *OtherRC_S_* in the card would be displayed on the doctor’s computer screen, as shown in Equation (8), for the doctor understanding the patient’s physical conditions.

(8) $$NHI\to DR\;OtherRC_{S}$$

After the doctor completes the diagnosis of the patient, the diagnosis content *NewRC_S_* is added to the patient’s medical records, as shown in Equation (9). Meanwhile, the patient’s medical record contents are stored in the database of the Bureau of National Health Insurance and transmitted, with the doctor’s group signature $Sig_{K_{D}}(NewRC_{S})$, to the Bureau of National Health Insurance for integration.

(9) $$DR\to NHI:NewRC_{S},\;Sig_{K_{D}}(NewRC_{S})$$

On the other hand, the doctor should fill in the prescription *R_X_*, including the number *R_X__ID* and the prescription content *MR_S_*. Similarly, the doctor’s group secret key *K_D_* is used for the signature. All data, including the patient’s public key certificate *PCert_P_*, encrypted (E) by the public key *PK_PH_* of the pharmacy (PH) designated by the patient, are transmitted, as shown in Equation (10).

(10) $$DR\to PH:E_{PK_{PH}}(R_{X},\;R_{X}\_ID,MR_{S},Sig_{K_{D}}(R_{X},\;R_{X}\_ID,MR_{S}),\;PCert_{P})$$

The latest diagnosis data on the patient’s national health insurance IC card is also updated, as shown in Equation (11), and the prescription number *R_X__ID* is enclosed for the authentication and signature when the patient collects medicine.

(11) $$DR\to P:NewRC_{S},\;R_{X}\_ID$$

When a patient needs to apply for insurance from an insurer (I), he/she merely needs to ask the doctor transforming the diagnosis content *NewRCS* into the diagnosis certificate *MC_S_* and the expense data *EP_S_*, with the doctor’s group secret key *K_D_*. It would integrate the patient’s insurance records. All data, containing the patient’s public key certificate *PCert_P_* and the designated insurer’s (I) public key *PK_I_* are encrypted (E) and transmitted, as shown in Equation (12).

(12) $$DR\to I:E_{PK_{I}}(MC_{S},\;EP_{S},Sig_{K_{D}}(MC_{S},\;EP_{S}),\;PCert_{P})$$

#### 3.2.2. Emergency

At this phase, a patient is delivered to a hospital emergency and is unconscious because of a major injury. The process is described as follows.

First, the patient, who is in coma, is delivered to emergency, and medical staff needs to acquire the fingerprint *FS* with a fingerprint identification machine. The doctor needs to insert the doctor card in the reader for the group signature $Sig_{K_{D}}(FS)$ of the fingerprint *FS*, which is encrypted (E) with the public key *PK_NHI_* from the Bureau of National Health Insurance and then transmitted, as shown in Equation (13).

(13) $$DR\to NHI:E_{PK_{NHI}}(FS,Sig_{K_{D}}(FS))$$

The machine would connect to the Bureau of National Health Insurance’s system, as shown in Equation (14), which would decrypt (D) with the personal, secret key *SK_NHI_*. The group signature public key *PK_GK_* is then used for verifying (*V*) the correctness of the doctor’s secret signature and substitutes the doctor card and the fingerprint *FS* data into the database of the Bureau of National Health Insurance to search for the patient’s data. The verification results with the group signature public key are stored in the machine in the Bureau of National Health Insurance to ensure the investigation of medical malpractice claims.

(14) $$\begin{array}{ll} DR↔NHI: & D_{SK_{NHI}}(E_{PK_{NHI}}(Sig_{K_{D}}(FS))),\\ & V_{PK_{GK}}(Sig_{K_{D}}(FS))\;\underline{\underline{?}}\;FS \end{array}$$

Once the verification is correct, and the fingerprint is confirmed, the patient’s basic data, medical records, and recent diagnosis records *OtherRC_S_* are displayed on the doctor’s computer screen, as shown in Equation (15), for the doctor understanding the patient’s physical conditions.

(15) $$NHI\to DR:OtherRC_{S}$$

### 3.3. Collecting Medicine Phase

A pharmacy would access the doctor’s group signature $Sig_{K_{D}}(R_{X},\;R_{X}\_ID,MR_{S})$ by encrypting (*D*) with personal, secret key *SK_PH_*, as shown in Equation (16), and verify (*V*) the correctness of the doctor’s prescription data with the group signature public key *PK_GK_* announced by the Bureau of National Health Insurance. The verification result is stored in the pharmacy machine to ensure the assistance of the Bureau of National Health Insurance in medical malpractice claims or monetary disputes.

(16) $$\begin{array}{ll} PH↔NHI: & D_{SK_{PH}}(E_{PK_{PH}}(Sig_{K_{D}}(R_{X},\;R_{X}\_ID,MR_{S}))),\\ & V_{PK_{GK}}(Sig_{K_{D}}(R_{X},\;R_{X}\_ID,MR_{S}))\;\underline{\underline{?}}\;R_{X},\;R_{X}\_ID,MR_{S} \end{array}$$

The patient then carries the national health insurance IC card to collect medicine in the pharmacy. The patient inserts the national health insurance IC card into the pharmacy machine, as shown in Equation (17), to judge the prescription number *R_X__ID* stored in the patient’s card conforming to the number transmitted from the doctor’s, and the pharmacy could confirm whether it is the patient collecting the medicine. The pharmacy gives the medicine to the patient and marks the prescription number as collected, to complete the entire process.

(17) $$\begin{array}{ll} P\to PH: & \;Card(R_{X}\_ID)\;\underline{\underline{?}}\;R_{X}\_ID,\\ & \;Sig_{SK_{P}}(R_{X}\_ID) \end{array}$$

Furthermore, an agent could collect the medicine as ruled, when the patient is disabled or in emergency. Accordingly, the agent could acquire the secret proxy key *SK_AP_* at the registration phase for collecting medicine. The agent could collect medicine by using the secret proxy key in the national health insurance IC card to sign $Sig_{SK_{AP}}(R_{X}\_ID)$ the prescription number, as shown in Equation (18).

(18) $$A\to PH:Sig_{SK_{AP}}(R_{X}\_ID)$$

The pharmacy would apply the patient’s public key *PK_P_* and the agent’s public key *PK_A_*, as shown in Equation (19), to verify (*V*) the correction of the prescription signature by returning the agent’s proxy public key *PK_AP_* through *F_A_* (to prove the patient authorization).

(19) $$\begin{array}{ll} A↔PH: & PK_{Ap}=F_{A}(PK_{P},PK_{A})\\ & V_{PK_{Ap}}(Sig_{SK_{Ap}}(R_{X}\_ID))\;\underline{\underline{?}}\;R_{X}\_ID \end{array}$$

## 4. Function Analysis

The integrity, convenience, and security of the proposed medical system should conform to the characteristics of the mobility of medical records, urgency of emergency medical care, function of medicine collection, protection of connection function and security, and avoidance of medicine conflicts. The following descriptions would prove the system satisfying the above requirements.

Mobility of Medicine Records: Along with the popularity of mobile devices, e.g., smart cards, mobile phones, and PDAs, the advance of development technology would be able to fulfill mobile medical records when storage space, computing capability, and memory size are enhanced. In terms of the proposed IMIS, the matched national health insurance IC card would be designed as the content mentioned in the article, with the functions of signature, encryption, and decryption, as well as being able to securely store relevant medical records in the card, whose portability satisfies the mobile function. Although similar smart cards are applied to current medical systems, some complicated verifications are required for accessing relevant medical records. It is believed to present great development space in the application of electronic medical care. Smart cards similar to the one proposed in this study would be used in e-medical systems, e-patient records, and e-prescriptions to fulfill mobile medicine records.

The urgency of emergency medical care: Medical care is a skill to strive for life. Although the advance of medical care technology enhances the cure rate, it is necessary to understand a patient’s physical conditions before the treatment. However, when a patient is unconscious and is delivered to a medical institution without any alerts, various examinations are required for understanding the physical conditions. In this case, the patient’s survival rate might be reduced. It is no longer time-consuming to realize patient’s conditions. With the proposed IMIS matched with a fingerprint identification system, designed as the content in this study, it would present the functions of signature and encryption/decryption and can retrieve a patient’s medical record data to satisfy the urgency of emergency medical care and show complete protection of information. Although some simple examinations could be done, finer data would effectively enhance the cure rate. The proposed emergency medical care would be really applied to establish a new milestone.

Medicine collection: Patients are given more convenience for medicine collection, especially for physically and mentally disabled patients. Legally authorizing an agent to collect medicine could prevent loss or fraud. Particularly, with fully electronic medical systems, medicine collection does not simply give a doctor signed prescriptions to a pharmacy. It becomes an important issue to provide secure medicine collection in the network environment full of people with bad intentions or computer hackers intercepting or revising prescription data. The IMIS applies proxy signature allowing an authorized agent to collect signature prescriptions, as well as maintain the signed digital signature with confirmation and integrity, avoiding forgery, and being undeniable so that the third verification party clearly knows the legality of patients and agents. In this case, it could guarantee a patient and an agent securely and actually collecting the medicine, as well as allow medical information system to provide medicine collection.

Protection of Privacy: Regarding the protection of privacy, the IMIS would ensure the five secure connection requirements through patients’ pseudonyms and doctors’ group signature, including anonymity, coalition-resistance, linkability of patients, linkability of doctors, and non-linkability of doctors. With anonymity, the pseudonym offered by the Bureau of National Health Insurance, real name, and privacy would not be disclosed. In this case, it could ensure patients’ clinical data privacy and provide academic units for research to further discover better treatment medicine or methods. With coalition-resistance, a doctor’s and a patient’s real names and privacy would not be disclosed by the cooperation of legal users. It could prevent patients with the same symptoms from being given test medicine by a pharmaceutical factory and avoid a pharmaceutical factory monopolizing the medicine for the symptoms to further effectively apply medical resources.

With patients’ linkability, after providing a patient with a pseudonym, the Bureau of National Health Insurance, could identify the relationship between the real identity and the pseudonym of a patient. Anyone (except the outpatient doctor) could not easily acquire the information. Meanwhile, a pharmacy could merely identify, with the same pseudonym, that different medical records belong to the same patient. Thus, the proposed IMIS could satisfy patients’ linkability. In terms of a doctor’s non-linkability, the use of group signature in IMIS merely allows just the Bureau of National Health Insurance tracing a doctor’s diagnosis signature and knowing the doctor’s real identity. Other units could merely verify the legality of the signature, but not the real identity of the doctor. Such a situation satisfies a doctor’s non-linkability that a pharmacy could not know the doctor from the medical records.

In addition, personalized medicine, also known as precision medicine, is a medical model that divides people into different groups, i.e., tailored medical decisions, practices, interventions, or products for patients based on their expected response or disease risk. In personalized medicine, diagnostic tests are usually used to select the appropriate and best treatment, based on the patient’s genetic composition, or other molecular or cellular analysis backgrounds. In such a situation, it is necessary to maintain a regular connection with the patient to ensure that tailored medical decisions can be made at any time during the treatment process. In our system, the patient’s linkability is used to ensure the connection of each different alias to a specific patient, while ensuring the connectivity of the attending physician. Therefore, there are no obstacles in the medical module in this area. It can find a more unified treatment for the individuals and their genome targeted by personalized medicine to develop better drugs and provide a better diagnoses for the targeted treatments.

Privacy of Insurance Companies: Using our system, the insurance company will receive the following benefits: (1) The received diagnosis certificate and fee receipt cannot be tampered with. (2) Relevant certificates issued by non-doctors cannot be sent or counterfeited. (3) Ensure the patient’s ability to pay the expenses. The detailed descriptions are as follows: In the process of transmission, as the transmitted data is signed by the doctor (medical office) with the private key of the group and encrypted by the public key of the insurance company, it ensures the security of the data cannot be tampered with, and the signature also makes the document undeniable. In addition, because the patient’s public key certificate, *PCert*_P_, is attached to the data, it can be used to ensure that the patient can make payment for the relevant expenses. Therefore, insurance companies can get these benefits and further prevent insurance fraudulence or repetitive claims, in turn, preventing major losses.

Shared Information between the medical office and health insurance companies: The information shared by the medical office (medical unit or doctor) and health insurance companies is the patient ID, diagnosis certificate, expense receipt. When a patient needs to apply for medical insurance from an insurance company, he/she only needs to ask the medical office to convert the contents of the diagnosis and treatment into the diagnosis certificate and expense receipt required for the insurance application and attach the doctor’s group private key signature to signify the doctor’s responsibility. Together with the patient’s public key certification, *PCert*_P_, it is encrypted with the public key of the insurance company designated by the patient and sent to the insurance company, which completes the transfer of information between the two units.

Security of all phases of system: The encryption mechanism and signature mechanism employed are all security systems that already exist and are implemented today (such as RSA public key mechanism; DSA, digital signature mechanism), and their security is based on their security certification and unsolvable math problems. Therefore, during the entire process, the patient will arrive at the hospital with an insurance IC card. The patient inserts the insurance IC card into the registration machine of the hospital to register and uses the personal, secret key to sign the latest timestamp (TS) on the computer. The hospital will check the validity of the patient’s health insurance payment data (Data). Then, in the outpatient clinic, the doctor and the patient insert the physician card and insurance IC card into the reader, respectively, and the relevant information will be encrypted and transmitted with the public key of the NHI. At this time, the reader will be connected to the NHI system, and the operator of the NHI party will use the secret key and the patient’s public key to decrypt the data to analyze the correctness of the relevant medical documents. Once the verification is correct and the patient ID is confirmed, the verification result will be stored in the NHI’s database to ensure that future medical disputes can be investigated; finally, the patient’s basic information and historical medical records will be returned, and the records of the recent diagnosis and treatment in the card are displayed on the doctor’s computer screen for the doctor to diagnose the patient’s physical condition. In the medicine collection phrase, the pharmacy obtains the group signature through a private key and verifies the correctness of the prescription information sent by the doctor. This verification result will be stored in the pharmacy to ensure that in the future, should there be any medical disputes or financial disputes, the stored information can be used for dispute resolution by NHI. Next, the patient will bring the insurance IC card to the pharmacy to collect the medicine; the pharmacy will determine whether the prescription number, *R_X__ID*, stored in the patient card matches the number transmitted by the hospital. If it is correct, the pharmacy will ask the patient to sign the number with a private key against the number to become the basis for the pharmacy to apply for subsidies for medical expenses from the NHI. Finally, the pharmacy gives the medicine to the patient and marks the prescription number as having received the medicine to complete the whole process. The above processes are all secured under the existing encryption mechanism and signature mechanism; thus, the privacy of patients and related medical institutions is guaranteed.

In terms of security of medical records collection, since the patient verification is issued by the NHI during the registration phase, this ensures that the patient is a legitimate user. When the patients go to the hospital for relevant physical examinations, the related medical records will be simultaneously written into the system. The process requires the matching of the IC card insertion of the medical staff and patients to read the private key for signature and use the NHI public.

## 5. Conclusions

An integrated medical information system with security policy and privacy protection, which combines e-patient records, e-prescriptions, modified smart cards, and fingerprint identification systems, and applies proxy signature and group signature, has been proposed in this study. The following conclusions are proposed.

E-Patient records could be exchanged in different medical institutions through the Bureau of National Health Insurance, and prescriptions and diagnosis certificates could be integrated and used. The use of personal electronic health records and electronic medical records could be very useful, and patients, doctors, hospitals, pharmacies, and pharmaceutical factories could adapt to this system. In addition to avoiding a pharmaceutical factory’s monopoly of medicine prices, medical institutions colluding with patients for diagnosis fees, and saving medical resources, contributing to environmental protections.

The medium of a fingerprint identification system could develop the urgency of emergency medical care. The fingerprint identification could confirm a patient’s identity and retrieve a patient’s electronic medical records saved in the Bureau of National Health Insurance for treating patients with major injuries or unconsciousness.

The functions of proxy signature and group signature could create secure verification and protection of doctors and patients. This would prevent patients from having to forgo treatment due to medical or privacy insecurity for immediate research on rare diseases and breakthroughs in medicine. Doctors, on the other hand, could make progress on the technology. There is no database of famous doctors who prescribe all the various medicines that exist within the medical field. Therefore, it might be discovered that certain medicines show better effectiveness when treating certain symptoms. It would simplify the complicated and time-consuming medical process.

The system would effectively apply and save medical resources—satisfying the mobility of medical records, presenting the function and security of medicine collection, and avoiding medical conflicts and profiteering to further acquire the maximum effectiveness with the least resources. When electronic medical care is gradually emphasized and becomes the mainstream, it is believed that the security and convenience of this system could be applicable to future medical environments.

## Figures and Tables

**Table 1:** Parameters defined and used in the paper.

Person	Secret Key	Public Key
patient	*SK_P_*	*PK_P_*
Agent	*SK_A_*	*PK_A_*
Bureau of National Health Insurance	*SK_NHI_*	*PK_NHI_*
doctors	*SK_D_*	
group signature	*K_D_*	*PK_GK_*
Patient proposes application for BNHI		*R_P_*
Detailed items to apply for health insurance		*Req*
Patient’s key certificate		*Cert_P_*
Serial number		*PID_P_*
Patient’s public key certificate		*PCert_P_*
Doctor’s key certificate		*Cert_D_*
Doctor’s application for group signature from BNHI		*R_D_*
Patient’s recent signature		*TS*
Recent diagnosis records		*OtherRC_S_*
Diagnosis content of the time		*NewRC_S_*
prescription		*R_X_*
Prescription content		*MR_S_*
Diagnosis certificate		*MC_S_*
Diagnosis expense data		*EP_S_*
Electronic payment details		*E-Payment*

## Data Availability

The data presented in this study are available on request from the corresponding author. The data are not publicly available due to further study will be carried out using the same data.
